# Progress in Surgery

**Published:** 1897-01-16

**Authors:** 


### PROGRESS IN SURGERY.

### DISEASES OF THE UPPER AIR PASSAGES.

*(Continued from page 252.)*

**Papillomatous Degeneration of the Nasal Mucosa.**—An interesting case of diffuse papillomatous degeneration is reported by Hunter Mackenzie[1], in a man aged 30, in whom the mucous membrane in both nostrils was studded throughout by numerous sessile growths varying in size from a pin's head to almost a grain of rice, the size of the great majority of them being about intermediate between these two extremes. They were most abundant on and about the upper regions (roof, outer wall, and septum), but many were located on and about the inferior areas of the nose. Both nostrils appeared to be equally affected. Several of the larger growths were removed with the cold snare, but the greater number could be detached only by means of the nasal curette. Four months afterwards there was no recurrence. The growths were examined microscopically by Dr. R. F. C. Leith, who reported as follows: that "microscopical examination showed the undoubted papillomatous character of the growths. . . . The conversion here seen of the columnar glandular epithelial covering of the nasal mucous membrane into cells exactly similar to those of the Malpighian layer of ordinary stratified squamous epithelium is very interesting."

The author states that nasal papillomata are of rare occurrence. The few cases hitherto recorded have generally been instances of single, less frequently of multiple, growths, and their histological characters have usually been rather of the fibrous than of the true papillomatous character. Hence the term "papillomatous fibroma," which it has been proposed to apply to them. In several instances they appear to have been simple hypertrophies of the nasal mucous membrane. Ordinary mucous polypi, it may be mentioned, occasionally develop papillomata on their surface, especially if they have been exposed to external irritation.

**Nasal Polypus.**—Delie[2] reports a case in a male patient who had suffered from nasal obstruction for more than twenty years. The nose was distended from root to tip. The mass of polypi in the left nostril weighed 40 grammes when removed, while those in the right nostril weighed 63 grammes, the whole together amounting to 103 grammes.

**Nasal Tuberculosis.**—Polyak[3] reports a case in a man, forty-nine years of age, who had complained of hæmorrhage and obstruction in the right nostril of six months' duration, and subsequent hæmorrhage and formation in the left nostril. The right nostril was completely filled by an irregular tumour resembling granulations, which bled easily. On the left side of the anterior portion of the septum was a tissue partly covered with dried crusts of a dull white, of the dimensions of a thaler-piece, flattened, granulated here and there, but not prominent, and bleeding easily. The tumour on the right side was at once removed with the incandescent snare, when it was found that the base of the neoplasm had occupied the right side of the septum at a point corresponding with the lesions described upon the left side. Slight hæmorrhage, consecutive to the operation, was arrested immediately by an insufflation of dermatol. The tumour removed was thirty millimetres in length, and forty millimetres in breadth, and had erosions upon the external surface where it was covered with granulations. It contained many tubercles and very few bacilli. There was no tuberculosis of the lungs or other portion of the body. The subsequent history of the case is unknown.

**Oxygen in the Treatment of Diseases of the Nose.**—The method of applying oxygen in the treatment of ulcers and various diseases has recently been described in The Hospital by George Stoker,[4] who devised the method about two years ago. In treating nasal affections he fills the bags, containing about one cubic foot, with equal parts of oxygen and pure air. The nose piece is passed into one nostril, the other nostril being plugged with cotton wool; the patient is directed to breathe through the mouth, the taps are turned on, and the treatment is begun. In ear cases the only difference is that the terminal piece is placed in the external auditory meatus, and in case of either ears or nose it is desirable to have several different sized terminals to fit different sized orifices. The oxygen should be allowed to pass into either the nose or ear from three to six hours daily. In nose cases it is best to use it about half an hour to an hour at a time, giving intervals of rest between the times. If used for more than an hour in nose cases it is apt to cause headache. The only additional treatment is using warm water to cleanse the parts during the day; the necessity for doing so seldom or often will, of course, depend on the amount of the discharge, but it must not be done less than twice daily.

He gives the notes of four cases, *i.e.*, two of "ozœna" and two of "suppurative middle-ear disease." These are typically bad cases, and seem to amply illustrate the results of the treatment.

**Adenoids.**—Jno. Wright,[5] working on the lines suggested by Dieulafoy's reports on concealed tuberculosis of the tonsils, has repeated the experiments of Dieulafoy in twelve unselected cases, inoculating guinea-pigs from tonsils and adenoids, and though each case was examined histologically and bacteriologically by W. H. Park, with negative results. As Strauss and others have found tubercle bacilli in healthy noses and throats, Wright thinks that Dieulafoy's results were due to surface contamination.

**Antral Empyema.**—A case of sero-purulent maxillary sinusitis due to lead poisoning, reported by H. L. Wagner,[6] is peculiarly interesting, since it proves how necessary it is to take a general and broad-minded view of patients complaining of affections, which at first appear definitely circumscribed and localised in their pathology. Wagner states that H. M., aged thirty-two — occupation, carriage-painter for twelve years—consulted him a year ago for severe neuralgia of the right supra-orbital region. These neuralgic pains had existed for three consecutive years, occurring daily at intervals from one to twelve hours, and consequently the patient was obliged to relinquish all work. He was afflicted with hyperosmia, strong odours of any kind producing pain. Opiates and various coal-tar derivatives were prescribed by his former physicians without any result, and the resection of the right supra-orbital nerve even failed to bring relief. All teeth in the upper jaw—some of them decayed and discoloured —had been extracted, the source of trouble being located there, but no relief followed.

*Examination.*—Skin yellow in colour, no syphilis; throat showed slight pharngitis sicca. Nose: Left side, normal; right, slight hypertrophy of the middle and lower turbinated bodies. At the entrance of the hiatus semilunaris a crust formed daily, which could easily be removed, and a slight sero-purulent discharge —containing staphylococcus aureus and a few non-pathogenic cocci—could only be observed every second or third day. Face: Transillumination showed little difference between the two sides. Pressure on the supra-orbital region produced no pain. Mouth: Hypertrophy of the right upper gum; no blue lead line could be detected. Pressing upon the region of the right first molar produced severe neuralgic pain. This assured him that the cause of the trouble existed in the right antrum. Being unable to probe or irrigate the antrum through the hiatus semilunaris, he entered the cavity through the hard palate under cocaine anæsthesia with the aid of a spearhead drill (this method, he says, is quick and painless, and he employs it frequently), and by injecting sterilised warm water a slight sero-purulent discharge was observed coming from the right nostril. Shortly after he decided to open the antrum through the canine fossa, with a large trephine drill. The antrum showed in the lower and side walls a peculiar bluish-grey hypertrophy of the mucous membrane. Probing did not reveal any caries of bone, but touching certain places produced severe pain. Microscopical examination of the hypertrophy, made by Dr. D. Montgomery, showed "loose connective tissue infiltrated with much serum and a fair number of round cells of inflammation; the piece of tissue had a covering of columnar epithelium. There were some micrococci in the tissue." After thorough removal of the hypertrophied tissue, dry treatment with borated gauze gave no relief, and also other methods of treatment were unsuccessful. He then examined the urine for albumen, sugar, and lead, none of which were found; but on examining fresh tissue, removed from the antrum, he found with sulphide of sodium the characteristic lead reaction. He placed the patient at once under iodide treatment, and in a few days he was relieved of all pain; the sero-purulent discharge then ceased, and with it the formation of crust. Traces of lead were then detected in the urine. Wagner assigns to lead poisoning the condition of the antrum, and neuritis of several nerves. Similar conditions, he states, have been observed in a few eye cases (Stood), where optic neuritis, accompanied by severe headache, was produced by lead poisoning.

**The Complications of Accessory sinus empyemas** are discussed by Lichwitz,[7] whose large experience renders such a summary of experience most valuable. Complications may involve (1) the neighbouring organs; (2) distant organs; (3) the general condition. Class I.—1. Nasal Fossæ: The mucous membrane of the turbinates may be atrophied, as in thirteen of the author's cases, and may simulate true ozœna. Three of his patients had been previously treated for ozœna, which he cured by treating the sphenoidal sinus. Six others had employed nasal douches, and had been cauterized; in these the nasal secretion diminished after the first irrigation of the sphenoidal sinus, and cephalalgia ceased entirely. In three other cases there seemed to be true ozœna with propagation to the sphenoidal sinus, and in the last case only maxillary sinusitis had been detected. Hypertrophic rhinitis, he states, is frequently met with. In one case there was also slight exophthalmos with less strabismus and diplopia and terrible cephalalgia. A malignant tumour of the superior maxilla was thought of. The patient, however, was cured by evacuation of the pus from the nose and antrum. In twelve cases he found small polypi or granulations over the semi-lunar hiatus, and in eighteen cases large mucous polypi, filling both sides of the nose, along with unilateral sinusitis. The polypi were probably rather the cause than the consequence of the empyema. Kakosmia was present in a third of his cases. 2. Naso-pharyngeal cavity and oral pharynx: Many of his patients, chiefly those with sphenoidal or frontal sinusitis, presented symptoms formerly described under the name of naso-pharyngeal catarrh, or Tornwaldt's disease. In many of his cases he has noted a complication of swelling and abscess of the palatine and pharyngeal tonsils. 3. Eyes: He has only observed the following complications in these cases—Dacryo-cystitis, three times, in two cases due to suppuration of several sinuses, and in one case to suppuration of the maxillary antrum; two cases of exophthalmos; and once he observed intense engorgement of the retinal veins in a patient who for twenty-four years had suffered from frontal sinusitis; they recovered their normal aspect when the empyema was cured. In two of his cases there was atrophy of the optic nerve, limited in one of his patients to a portion only of the retina; this patient had suppuration of the frontal sinus with eruption of the pus into the orbit. In the other case it was probably due to sphenoidal sinusitis. Once he noticed papillary inequality in a case of sphenoidal sinusitis. 4. Ears: He has observed fifteen cases of old or recent suppurative otitis, once with mastoiditis; seven times sub-acute otitis; fourteen times chronic median otitis, with or without Eustachian obstruction; nine times nervous buzzings; and three times vertigo. 5. Cranial-facial complications: The most frequent is cephalalgia, generally when the sinusitis is sphenoidal or frontal. Four times he has found facial neuralgia with maxillary sinusitis. In one of his cases irrigation of the right sphenoidal sinus caused epileptiform crisis, with loss of consciousness. 6. Cutaneous affections of the face: Five patients had recurrent erysipelas; he believes it to be a consequence and not a cause of sinusitis. Nasal eczema and acne rosacea have been observed by him in several cases. He has seen erythema and fugitive œdema after injection of antiseptic fluids. Class II.—7. Bronchial complications: He has often seen acute or sub-acute laryngeal catarrh, twice with thickening of the cords, and many times with pareses of the glottic constrictors. Bronchitis is caused sometimes, probably by penetration of pus into the bronchi, especially during sleep. Sometimes rebellious bronchorrhœa follows. One of his patients had signs of a pulmonary abscess or lobular pneumonia. Three times he noted cough, disappearing only after evacuation of the sinus; five patients had symptoms resembling asthma. 8. Four times he found dyspepsia, and three times rebellious diarrhœa alternating with constipation. 9. Vascular and cardiac affections: One of his patients, with empyema of all the sinuses, had a slow pulse, 28-30 to the minute, which became normal after treatment. In two patients with old maxillary sinusitis he found two attacks of phlebitis to have occurred. Class III.—General affections: He has noted general enfeeblement and loss of flesh ten times, fever four times, insomnia three times, unconquerable somnolence three times, aprosexia twice, cerebrasthenia several times, and pronounced melancholia four times. Crises resembling *petit mal*, in a boy of ten, were cured after removal of the pus from the sphenoidal sinuses.

Treatment: He prefers opening into the maxillary sinus through the alveolar apophysis or canine fossa. For the frontal and sphenoidal sinuses, irrigation through the natural orifices has always been sufficient for him, though he has often had to remove the middle turbinated and enlarge the orifices. Wherever it is possible, he prefers the endo-nasal treatment.

[1] Lancet, 1896. [2] Journ. of Laryng., Oct., 1896. [3] Rev. Hebd. de Laryng. d'Otol. et du Rhin., No. 4, 1896; Amer. Journ. of the Med. Sci., Aug., 1896. [4] New York Med. Journ., Aug. 29, 1896. [5] Trans. of Amer. Laryng. Assoc.; Journ. of Laryng., Aug., 1896. [6] New York Med. Journ., Aug. 15, 1896. [7] Trans. by Wolfenden in Journ. of Laryng., 1896.

